# Supplementary material for: Evidence supported by Mendelian randomization: impact on inflammatory factors in knee osteoarthritis
Source: Front Med (Lausanne). 2024 May 28;11:1382836. doi: 10.3389/fmed.2024.1382836 (PMC11165061; doi:10.3389/fmed.2024.1382836)
Supplement: Supplementary file 1 [file Data_Sheet_1.ZIP › data/GCST90274825_5e6/825.forest.pdf]

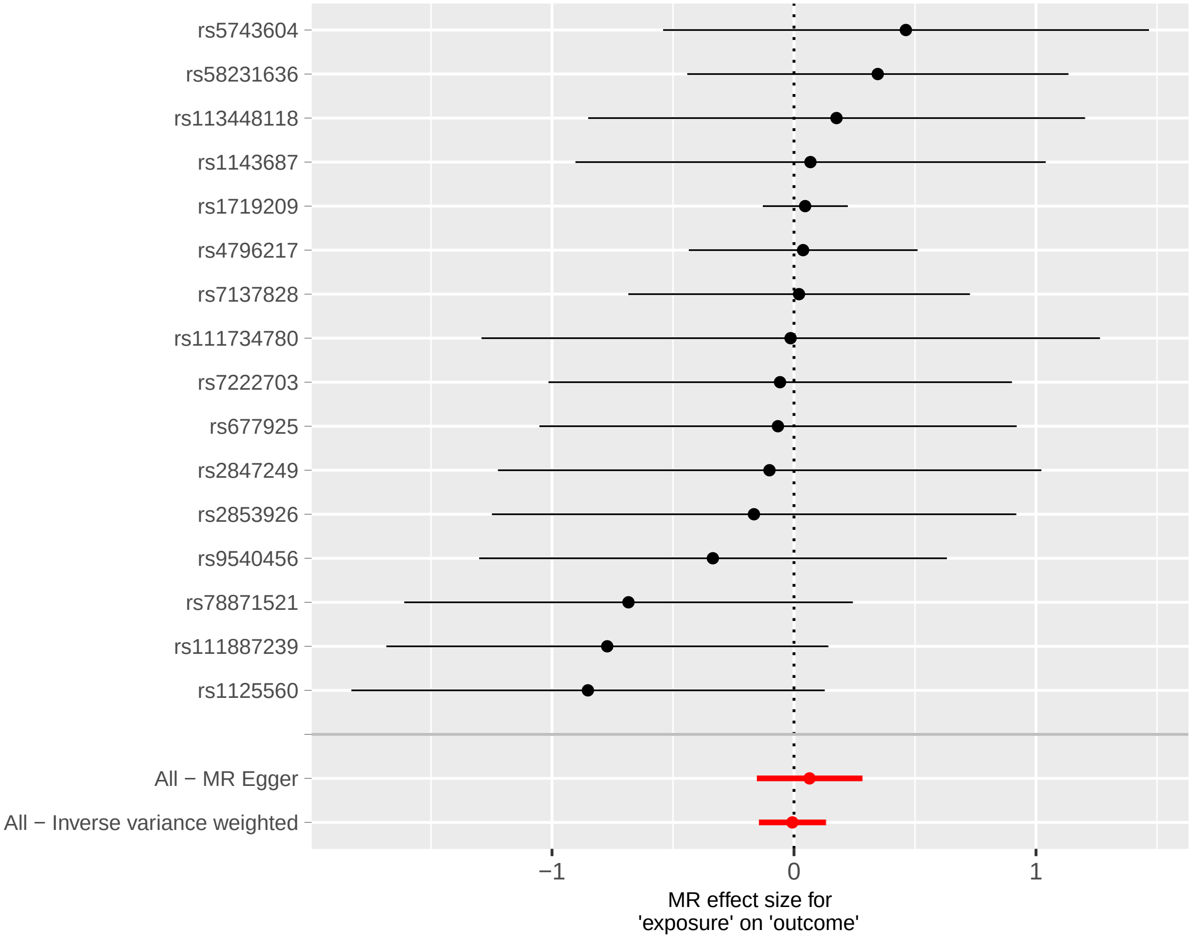
rs5743604
rs58231636
rs113448118
rs1143687
rs1719209
rs4796217
rs7137828
rs111734780
rs7222703
rs677925
rs2847249
rs2853926
rs9540456
rs78871521
rs111887239
rs1125560
All − MR Egger
All − Inverse variance weighted
−1
0
1
MR effect size for
'exposure' on 'outcome'
